# Thai rice instant granules containing turmeric extract and *Phyllanthus emblica* fruit pulp: Chronic toxicity and antioxidant profiles in rats and *in silico* investigation of bioactive compounds

**DOI:** 10.3389/ftox.2025.1691014

**Published:** 2025-12-05

**Authors:** Charatda Punvittayagul, Atigan Thongtharb, Sonthaya Umsumarng, Siripat Chaichit, Siriporn Okonogi, Chadarat Ampasavate, Darunee Hongwiset, Sirinya Taya

**Affiliations:** 1 Center of Veterinary Medical Diagnostic and Animal Health Innovation, Faculty of Veterinary Medicine, Chiang Mai University, Chiang Mai, Thailand; 2 Faculty of Veterinary Medicine, Chiang Mai University, Chiang Mai, Thailand; 3 Department of Pharmaceutical Sciences, Faculty of Pharmacy, Chiang Mai University, Chiang Mai, Thailand; 4 Multidisciplinary Research Institute, Chiang Mai University, Chiang Mai, Thailand

**Keywords:** bioactive compounds, chronic toxicity, curcumin, Indian gooseberry, *Oryza sativa*, *Phyllanthus emblica*, Thai rice, turmeric

## Abstract

Turmeric (*Curcuma longa*) and Indian gooseberry (*Phyllanthus emblica*) are widely used for their ethnopharmacological properties, particularly in ASEAN countries. Recently, our group has formulated Thai rice instant granules containing turmeric extract and *P. emblica* fruit pulp (TR instant granules); however, their toxicity profile has not been evaluated. This study investigated the long-term safety of TR instant granules in rats and assessed their effect on hepatic antioxidant status. Oral administration at doses of 200, 600, and 2,000 mg/kg body weight for 180 days resulted in no toxic effects, behavioral changes, mortality, or alterations in organ weights or hematological parameters. Significant changes were observed in biochemical markers, but there were no histopathological abnormalities. TR instant granules significantly upregulated hepatic antioxidant genes (*Nrf2, GPx, CAT, GR, SOD, and HO-1*). Phytochemical analysis identified chlorogenic acid and curcumin as major constituents. In silico molecular docking demonstrated that these compounds strongly bind multiple antioxidant enzymes and the Nrf2–Keap1 complex, supporting their potential as effective modulators of cellular antioxidant defenses. Overall, long-term administration of TR instant granules is safe and may enhance antioxidant mechanisms in rats. These findings support the potential development of TR instant granules as a safe functional food or nutraceutical with antioxidant benefits.

## 1 Introduction

Traditional medicine is an essential part of healthcare systems, offering various practices and treatments developed over generations, particularly in China and the ASEAN countries. Much literature related to herbal and traditional medicine is becoming increasingly significant, attracting attention from researchers, wellness practitioners, and consumers worldwide (Yuan et al., 2016; Liu, 2021). Medicinal plants contain various bioactive compounds found in their roots, leaves, flowers, seeds, and fruits, which are believed to possess therapeutic properties (Sofowora et al., 2013; Ansari et al., 2025). Consequently, the study of medicinal plants and their bioactive constituents has gained considerable attention, especially for the development of new drugs and pharmacological products.

Turmeric (*Curcuma longa*) is a perennial herbaceous plant with rhizomes that is widely used in traditional medicine due to its numerous therapeutic benefits (Doyle et al., 2023). It has origins in India and is distributed and cultivated in several countries. Chinese traditional medicine utilizes turmeric to treat and prevent gastric ulcers, inflammation, cancer, and other diseases (Iweala et al., 2023; Tung et al., 2019). Turmeric rhizomes are composed of carbohydrates (approximately 69%) and 3%–5% phenolic compounds, including curcumin, demethoxycurcumin, and bisdemethoxycurcumin (Phipps et al., 2020). Among these, curcumin is the most abundant and possesses anti-inflammatory, antioxidant, and anticancer activities. However, its oral bioavailability is poor due to low absorption, rapid metabolism, and rapid excretion, as documented in numerous studies (Phipps et al., 2020; Vollono et al., 2019; Kocaadam and Şanlier, 2017; Soleimani et al., 2018).

Indian gooseberry (*Phyllanthus emblica*), a fruit native to India, is rich in phytochemicals that contribute to its therapeutic and nutritional benefits (Avinash et al., 2024). The major bioactive constituents found in the fruit include ascorbic acid (70%–72%) and polyphenols, such as gallic acid (approximately 5%), chlorogenic acid, ellagic acid, quercetin, luteolin, and naringenin (Khalid et al., 2022; Saini et al., 2022; Prananda et al., 2023; Gul et al., 2022). These are highly variable in their bioavailability based on their chemical structures. Ascorbic acid is water-soluble and is well absorbed by the gastrointestinal tract. In contrast, polyphenols such as gallic acid and ellagic acid exhibit low bioavailability because they undergo extensive metabolism in the liver and by the gut microbiota. Many reports have documented the biological properties of the fruit part, including anti-inflammatory properties, supporting digestion, preventing constipation, and balancing stomach acids. Indian traditional medicine uses this plant for treating cancer, diabetes, and cardiac problems (Saini et al., 2022; Prananda et al., 2023; Kumar et al., 2022).

Considering the pharmacological properties and traditional use of turmeric and *P. emblica* for promoting gastrointestinal health, their combination could produce synergistic or complementary therapeutic effects. Based on this reasoning, our team developed Thai rice instant granules (TR instant granules) containing turmeric extract and *P. emblica* fruit pulp. Granulation was employed to enhance the solubility and absorption of active substances, using Thai rice as a filler and binder due to its safety and value as a local ingredient. We hypothesized that combining these herbal components would not only improve their therapeutic potential but also help preserve the stability of curcumin (from turmeric extract) and other active compounds from *P. emblica* within the granule matrix. Therefore, chronic toxicity studies were necessary to assess the long-term safety of this combined formulation before clinical use or widespread application.

In general, toxicology testing is essential for pharmacological research and drug development (Denny and Stewart, 2017). To ensure the safety of long-term drug use, chronic toxicity studies are required to determine the safety of compounds before clinical studies and human use (Sachana and Hargreaves, 2018). Numerous studies have documented the safety and acute or subchronic toxicity profiles of turmeric extract and *P. emblica*. For instance, oral administration of *C. longa* extract at 3,000 mg/kg body weight (BW) was non-toxic, and subchronic toxicity at 100 mg/kg BW was also safe (Soleimani et al., 2018; Qureshi et al., 1992). However, no reports have been published on the chronic toxicity of *C. longa* extract in rats. Similarly, the *P. emblica* fruit extract has also shown a median lethal dose (LD_50_) greater than 5,000 mg/kg BW, and chronic treatment of the extract for 270 days in rats did not show any sign of toxicity (Saini et al., 2022; Jaijoy et al., 2011). However, most of the previous studies have focused on the toxic effects of these plants individually or on short-term toxicity. There is a lack of data on the long-term safety of their combination in a formulated product. Additionally, no reports have been published on the chronic toxicity of *C. longa* extract in rats. Therefore, this study aimed to evaluate the chronic toxicity of TR instant granules containing turmeric extract and *P. emblica* fruit pulp in rats, as well as their effects on hepatic antioxidant status. Molecular docking analysis was conducted to examine the binding interactions between major bioactive compounds in TR instant granules and key proteins involved in antioxidant pathways. This work represents the chronic toxicity assessment of TR instant granules, combined with *in silico* predictions of their antioxidant potential. It provides a foundation for future therapeutic applications and investigations.

## 2 Materials and methods

### 2.1 Plant materials and preparation of the granules

Turmeric extract was obtained from Thai-China Flavours and Fragrances Industry Co., Ltd. (TCFF), Ayutthaya, Thailand. Fresh *P. emblica* fruits were purchased from the local market and processed into powder after removing the seeds and allowing sufficient drying time at 60 °C. Thai rice (*Oryza sativa*) was obtained from the Lanna Rice Research Center, Chiang Mai University, Chiang Mai, Thailand.

The granules were prepared using the wet granulation method as described by Prakatthagomol and Okonogi (2012), with slight modifications (Prakatthagomol and Okonogi, 2012). The ingredients were combined by mixing 60% Thai rice, 20% turmeric extract, and 20% dried *P. emblica* fruit pulp powder. A suitable amount of binder was added to the mixture, which was then subjected to a high-shear mixer and granulator. The obtained wet granules were dried in a 40 °C oven until the moisture content was less than 5%. After drying, the granules were sieved using a 14-mesh sieve for particle control and used for experiments.

### 2.2 Evaluation of physicochemical properties and chemical constituents of Thai rice instant granules

#### 2.2.1 Morphology and physicochemical properties

The morphology of the TR instant granules was assessed through visual inspection. After drying and sieving through a 14-mesh sieve, the granules were collected, photographed, and evaluated without magnification. For pH determination, 80 mg of the granules were dispersed in 10 mL of distilled water and sonicated at 30 °C for 2 min. The pH of the resulting dispersion was measured using a pH meter (Metrohm, Model 691, Herisau, Switzerland). The loss on drying was determined using a moisture analyzer at 105 °C.

#### 2.2.2 Identification and quantitative analysis of active constituents in Thai rice instant granules

A high-performance liquid chromatography instrument (HPLC: Agilent Infinity 1260, Agilent (Agilent, United States), equipped with a quaternary pump, an autosampler, a column thermostat, and a photodiode array detector (DAD), was used for identification and quantification of the curcuminoids in TR instant granules. The separation was achieved on a Mightysil RP18 column (250 mm × 4.6 mm x 5.0 µm; Kanto Chemical, Japan). The mobile phase consisted of acetonitrile (A) and 0.5% v/v acetic acid (B) with a gradient elution as follows: 0–5 min isocratic elution of 10% A, 5–10 min linear gradient to 50% A, 10–30 min isocratic elution of 50% A and then 30–32 min dropped back down to 10% A. Post-run equilibration was 10 min. The autosampler and column temperatures were controlled at 20 °C and 30 °C, respectively. The flow rate was 1.0 mL/min. A wavelength of 425 nm was used for the identification and quantification of curcumin in the preparation.

Polyphenolic compounds in the TR instant granules were analyzed by analytical liquid chromatography using an Agilent 1260 Infinity II series chromatograph coupled with an Agilent 6130 electrospray ionization quadrupole mass spectrometer (Agilent Technologies, Santa Clara, CA, United States). The chromatographic separation was performed using an Ultra C18 column (5 μm 4.6 × 250 mm; Restek, Bellefonte, PA, United States) under the same conditions as described by Wisetkomolmat et al. (2022), and spectra were acquired in negative ion mode (Wisetkomolmat et al., 2022).

### 2.3 Animals and experimental protocol for the chronic toxicity test

#### 2.3.1 Animals

Male and female Wistar rats (n = 100; Male = 50, Female = 50), 8 weeks of age (100–110 g), were purchased from Nomura Siam International Co., Ltd., Thailand. The animals were acclimated for 1 week before the experiment under the laboratory conditions (a temperature of 22 °C ± 2 °C, relative humidity of 55% ± 10%, and an artificial 12:12 h light/dark regimen). Food and water were available *ad libitum*. The treatment protocol was approved by the Animal Care and Use Committee, Chiang Mai University (Protocol No. 2566/RT-0014, approved on 4 August 2023). The study was performed in accordance with the institutional guidelines. This study follows the recommendation in the ARRIVE guidelines.

#### 2.3.2 Experimental protocol

**FIGURE 1:**
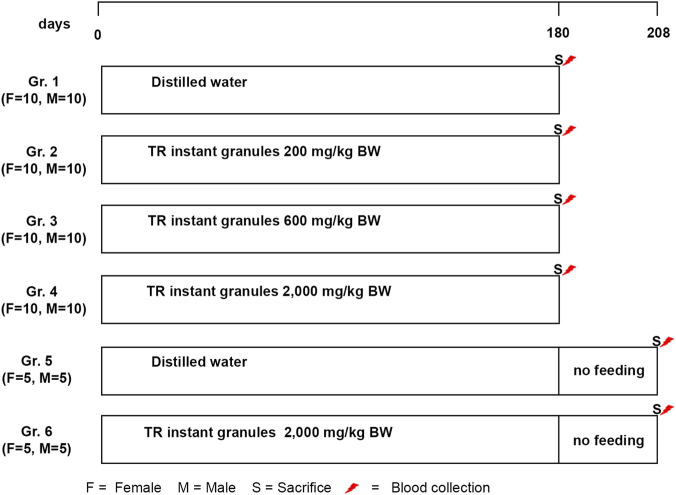
Treatment protocol for chronic toxicity study.

For the evaluation of chronic toxicity, the maximum dose was set at 2,000 mg/kg due to solubility limitations. The concentrations of 200 mg/kg and 600 mg/kg were selected as the low and medium doses, respectively, with each dose level approximately threefold apart. Chronic toxicity studies were conducted on female and male Wistar rats, following the Organization for Economic Co-operation and Development (OECD) test guideline No. 452 (OECD, 2018), with some modifications. Rats were randomly assigned to six groups. The main groups (Groups 1–4), each consisting of ten males and ten females, received testing substances orally through a feeding tube for 180 days. Group 1 served as the control and was given distilled water. Groups 2–4 were orally administered TR instant granules at doses of 200, 600, and 2,000 mg/kg of body weight (BW), respectively. The satellite groups (5 males and 5 females each) were fed distilled water (Group 5) and TR instant granules at doses of 2,000 mg/kg BW daily (Group 6) for 180 days, followed by a 28-day treatment-free period. The satellite groups were established to observe recovery and assess the delayed effects of the TR instant granules. The treatment protocol is shown in Figure 1. The animals were monitored daily for signs of toxicity, physiological and behavioral changes, and mortality. Body weight, food consumption, and water intake were recorded weekly. At the end of the experiment, the animals were euthanized with an overdose of isoflurane after 16 h overnight fast. Blood samples were collected from the abdominal vein for hematological and biochemical analyses. The internal organs were excised, observed, weighed, and then fixed in a 10% neutral buffered formaldehyde solution for histopathological analysis. A portion of the male rat liver tissues was stored at −80 °C to study the effect of TR instant granules on gene expression.

#### 2.3.3 Hematological and biochemical blood analysis

Blood samples collected from male and female rats were sent to the hematology laboratory at The Small Animal Hospital, Chiang Mai University, Thailand, for hematological and biochemical blood analysis.

The hematological parameters analyzed included total and differential white blood cell (WBC) count, red blood cell (RBC) count, hemoglobin (Hb), hematocrit (HCT), mean corpuscular volume (MCV), mean corpuscular hemoglobin (MCH), mean corpuscular hemoglobin concentration (MCHC), platelet count (PLT), and mean platelet volume (MPV).

The biochemical analysis was performed to determine the following parameters: cholesterol, blood urea nitrogen (BUN), creatinine, total protein, albumin, alanine aspartate aminotransferase (AST), alanine aminotransferase (ALT), alkaline phosphatase (ALP), total bilirubin, and direct bilirubin. The electrolytes, including sodium ion concentration (Na^+^), potassium ion concentration (K^+^), chloride ion concentration (Cl^−^), and total carbon dioxide concentration (TCO_2_), were analyzed.

#### 2.3.4 Histopathological analysis

Fixed tissues were routinely processed for paraffin embedding, sectioned, and stained with hematoxylin and eosin according to standard histological procedures as described by Suvarna et al. (2019). The pathologist performed the histopathological examination and recorded the findings under a light microscope (×200 and ×400) for any lesions or abnormalities. Histopathological findings were evaluated using standard diagnostic criteria.

#### 2.3.5 Gene expression analysis by real-time polymerase chain reaction (RT-PCR)

This study aimed to evaluate the effects of TR instant granules on the expression of antioxidant genes after a 180-day treatment period. To reduce biological variability and prevent hormonal fluctuations associated with the female estrous cycle, which have a potential impact on oxidative stress and antioxidant enzyme activity, only male rats were used in this study.

The total RNA was extracted from the rat livers and converted into complementary DNA (cDNA), following the previously published report (Punvittayagul et al., 2022). The specific primers used in the study are listed in Supplementary Table S1. The PCR conditions were conducted following our previous research, utilizing the SensiFast™ SYBR Lo-ROX Kit (Bioline, France) (Punvittayagul et al., 2022). β-actin served as the internal control, and the relative fold expression of the genes was calculated using the 2^−ΔΔCT^ method.

### 2.4 In silico study

#### 2.4.1 Molecular docking study

The three-dimensional structures of the bioactive compounds found in TR instant granules (curcumin, desmethoxycurcumin, bisdemethoxycurcumin, chlorogenic acid, phytic acid, and gallic acid) were retrieved from the PubChem database (https://pubchem.ncbi.nlm.nih.gov/). These structures were geometry-optimized using Gaussian09w software, with the B3LYP/6-31G (d,p) basis set to achieve a stable conformation (Hehre et al., 1970; Frisch, 2009). Hydrogen atoms and Gasteiger charges were assigned using AutoDockTools 1.5.7 (Morris et al., 2009).

The human protein structures of target proteins related to antioxidant enzymes (Nrf-2, CAT, SOD, GPx, GST, GR, and HO-1) were obtained from the Protein Data Bank (PDB) database. All non-structural water molecules and co-crystallized ligands were removed from the structures to ensure accurate binding prediction. Hydrogen atoms and Kollman charges were applied to the protein structures using AutoDockTools 1.5.7.

The molecular docking simulations were performed using AutoDock Vina to predict the binding affinity (Trott and Olson, 2010). The binding pocket coordinates were set based on the known active sites of each target. The ligand conformation with the highest binding affinity (kcal/mol) was selected to analyze protein–ligand interactions and binding patterns. Visualization and interaction analyses were performed using Discovery Studio Visualizer 2017 (BIOVIA, 2017).

#### 2.4.2 Clustering and principal component analysis of bioactive compounds

To evaluate the binding affinity of the bioactive compounds, clustering analysis was conducted based on the binding energy to categorize the most potent compounds. Additionally, principal component analysis (PCA) was employed to identify compounds with the highest potential for modulating antioxidant and anti-inflammatory activities. Both clustering and PCA analyses were performed using the R program (Team, 2020), utilizing the ComplexHeatmap and the factoextra packages for visualization and statistical interpretation.

### 2.5 Statistical analysis

For chronic toxicity parameters and gene expression analysis, a one-way analysis of variance (ANOVA) and Dunnett’s test were conducted to assess the statistical significance of the main group. The independent sample t-test evaluated the significant difference among the satellite groups. Results are reported as mean ± standard deviation (SD). *P* values less than 0.05 were considered statistically significant. The statistical analyses were carried out using statistical software SPSS 21 (IBM, New York, United States).

## 3 Results

### 3.1 Morphology and physicochemical properties

**FIGURE 2:**
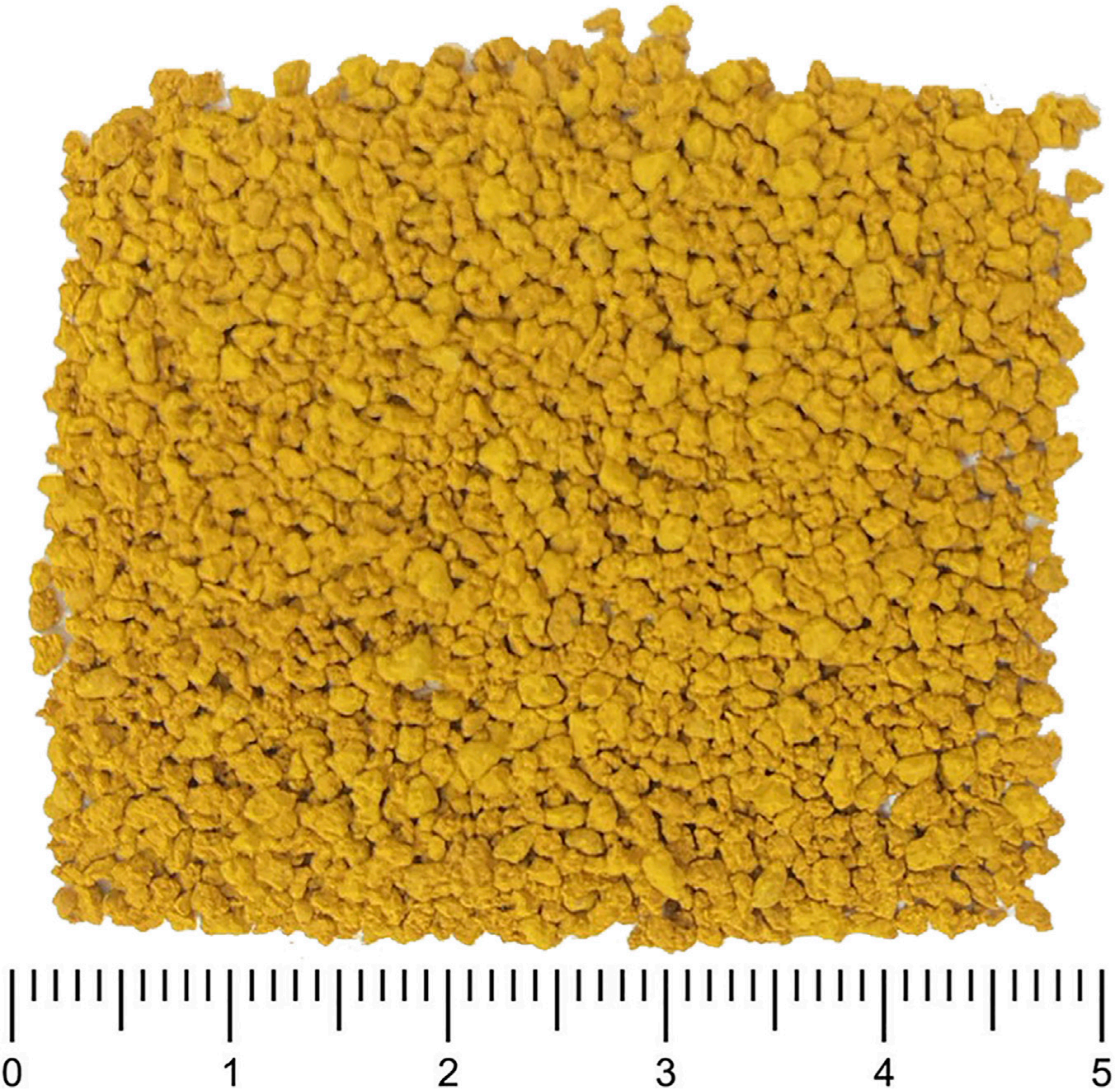
The morphology of the Thai rice instant granules.

The TR instant granules were round and mustard yellow (Figure 2). The granule sizes were controlled by sieving through a 14-mesh sieve. After being dissolved in DI water, the pH of the preparation was approximately 3.439 ± 0.004. The loss on drying (LOD) of the preparation was 3.04% ± 0.23%.

### 3.2 Identification and quantification of active constituents in Thai rice instant granules

Representative chromatograms of TR instant granules are shown in Supplementary Figure S1. Three distinct peaks at 19.4, 20.5, and 21.6 min, detected at 425 nm, correspond to the characteristic curcuminoid profile of turmeric. Curcumin quantification was performed using a validated analytical method following ICH and AOAC guidelines (Walfish, 2006; Pustaka et al., 2005). System suitability and validation parameters were evaluated over a concentration range of 5–15 μg/mL. The method demonstrated acceptable performance for the analysis of curcumin in the formulation, exhibiting linearity with an *R* ^2^ value greater than 0.99. The retention time of curcumin was approximately 22 min. The peak symmetric factor was 0.71 ± 0.01, and the theoretical plate count was 51255 ± 1257. Using this validated method, the curcumin content in the granule formulation was found to be 167.9 ± 3.3 mg/g sample.

The polyphenolic compounds in TR instant granules are summarized in Table 1. Eight phenolic compounds were identified by comparing their mass spectra and retention times with those of available standards. Chlorogenic acid exhibited the highest concentration at 62.05 ± 0.12 mg/g sample, followed by phytic acid (17.25 ± 0.66 mg/g) and gallic acid (11.26 ± 0.30 mg/g). Quercetin (10.50 ± 0.16 mg/g), rosmarinic acid (6.33 ± 0.027 mg/g), epicatechin (5.79 ± 0.052 mg/g), and caffeic acid (5.21 ± 0.072 mg/g) were also detected, while *p*-coumaric acid showed the lowest content at 4.78 ± 0.009 mg/g. Trans-ferulic acid, rutin, and naringin were not detected in TR instant granules.

**TABLE 1:** Polyphenolic compounds in Thai rice instant granules.

Analytes	mg/g sample
Gallic acid	11.26 ± 0.30
Epicatechin	5.79 ± 0.05
Caffeic acid	5.21 ± 0.07
*p*-Coumaric acid	4.78 ± 0.01
Trans-ferulic acid	ND
Rutin	ND
Rosmarinic acid	6.33 ± 0.03
Quercetin	10.50 ± 0.16
Naringin	ND
Phytic acid	17.25 ± 0.66
Chlorogenic acid	62.05 ± 0.12

Values are Mean ± SD (n = 2). ND, Not detectable. Detection limit of compounds: trans-ferulic acid, 0.088 mg/g; rutin, 0.107 mg/g; and naringin, 0.014 mg/g.

### 3.3 Chronic toxicity effect of TR instant granules in rats

#### 3.3.1 General observations and organ weights

There were no signs of toxicity or mortality in rats treated with TR instant granules for 180 consecutive days. Throughout the study period, all treated rats in each dosage group continued to gain weight normally. Additionally, there were no differences in food consumption or water intake in both male and female rats across the different dosage groups. These findings are presented in Supplementary Table S2.

Rat organs, including the liver, spleen, kidney, lung, heart, thymus, pancreas, brain, stomach, and adrenal gland, were all weighed in the study. The weight measurements of reproductive organs in males (testis, prostate, seminal vesicles, epididymis) and females (ovary, uterine horn and uterus) were also evaluated. The results are shown in Table 2. Male rats treated with TR instant granules exhibited a significantly greater weight increase in the spleen (600 and 2,000 mg/kg BW), liver (2,000 mg/kg BW), and lung (2,000 mg/kg BW). In female rats treated with TR instant granules, a significant increase in liver weight (2,000 mg/kg BW) was observed.

**TABLE 2:** Relative organ weight (%) of rats receiving Thai rice instant-granules containing turmeric extract and *Phyllanthus emblica* fruit pulp in the chronic toxicity test.

Relative organ weight (%)	Control	Main group			Satellite group	
		200 mg/kg	600 mg/kg	2000 mg/kg	Control	2000 mg/kg
Males						
Liver	2.79 ± 0.16	2.79 ± 0.31	2.88 ± 0.21	3.22 ± 0.20^a^	2.75 ± 0.25	2.86 ± 0.13
Spleen	0.13 ± 0.02	0.14 ± 0.01	0.14 ± 0.02	0.13 ± 0.01	0.14 ± 0.01	0.13 ± 0.01
Kidney	0.55 ± 0.05	0.55 ± 0.07	0.57 ± 0.08	0.61 ± 0.06	0.54 ± 0.06	0.54 ± 0.03
Lung	0.26 ± 0.07	0.27 ± 0.02	0.28 ± 0.03	0.31 ± 0.04^a^	0.27 ± 0.02	0.30 ± 0.02
Heart	0.22 ± 0.02	0.21 ± 0.02	0.21 ± 0.01	0.23 ± 0.02	0.20 ± 0.01	0.21 ± 0.01
Thymus	0.06 ± 0.02	0.07 ± 0.01	0.06 ± 0.03	0.08 ± 0.03	0.06 ± 0.02	0.07 ± 0.02
Pancreas	0.16 ± 0.03	0.17 ± 0.05	0.22 ± 0.05^a^	0.21 ± 0.05^a^	0.20 ± 0.05	0.17 ± 0.04
Brain	0.29 ± 0.03	0.30 ± 0.03	0.32 ± 0.05	0.31 ± 0.03	0.32 ± 0.05	0.32 ± 0.02
Testis	0.65 ± 0.05	0.67 ± 0.06	0.65 ± 0.07	0.67 ± 0.08	0.65 ± 0.02	0.63 ± 0.04
Prostate	0.11 ± 0.02	0.11 ± 0.03	0.11 ± 0.03	0.11 ± 0.03	0.09 ± 0.03	0.12 ± 0.02
Stomach	0.35 ± 0.02	0.35 ± 0.03	0.33 ± 0.04	0.37 ± 0.03	0.34 ± 0.02	0.34 ± 0.03
Seminal vesicle	0.25 ± 0.02	0.24 ± 0.03	0.25 ± 0.05	0.26 ± 0.03	0.26 ± 0.02	0.26 ± 0.04
Epididymis	0.29 ± 0.02	0.29 ± 0.05	0.30 ± 0.07	0.30 ± 0.08	0.28 ± 0.06	0.22 ± 0.06
Adrenal gland	0.010 ± 0.002	0.011 ± 0.002	0.013 ± 0.004	0.011 ± 0.003	0.012 ± 0.004	0.012 ± 0.002
Females						
Liver	2.58 ± 0.20	2.65 ± 0.12	2.76 ± 0.19	3.00 ± 0.27^a^	2.77 ± 0.32	2.81 ± 0.37
Spleen	0.17 ± 0.03	0.16 ± 0.02	0.16 ± 0.02	0.17 ± 0.02	0.18 ± 0.02	0.17 ± 0.03
Kidney	0.60 ± 0.07	0.60 ± 0.06	0.63 ± 0.06	0.68 ± 0.05	0.62 ± 0.06	0.64 ± 0.04
Lung	0.35 ± 0.03	0.37 ± 0.06	0.38 ± 0.06	0.39 ± 0.02	0.37 ± 0.04	0.38 ± 0.02
Heart	0.24 ± 0.02	0.24 ± 0.02	0.25 ± 0.02	0.26 ± 0.02	0.24 ± 0.03	0.24 ± 0.02
Thymus	0.10 ± 0.03	0.11 ± 0.02	0.11 ± 0.03	0.10 ± 0.03	0.08 ± 0.06	0.10 ± 0.03
Pancreas	0.25 ± 0.07	0.25 ± 0.09	0.26 ± 0.07	0.29 ± 0.08	0.26 ± 0.08	0.21 ± 0.04
Brain	0.55 ± 0.06	0.54 ± 0.05	0.55 ± 0.08	0.57 ± 0.05	0.52 ± 0.06	0.57 ± 0.07
Ovary	0.05 ± 0.01	0.04 ± 0.01	0.05 ± 0.01	0.05 ± 0.01	0.05 ± 0.01	0.05 ± 0.02
Uterine horn and uterus	0.21 ± 0.07	0.20 ± 0.03	0.22 ± 0.12	0.19 ± 0.05	0.21 ± 0.04	0.21 ± 0.07
Stomach	0.46 ± 0.03	0.45 ± 0.06	0.47 ± 0.05	0.48 ± 0.05	0.43 ± 0.06	0.48 ± 0.05
Adrenal gland	0.023 ± 0.006	0.023 ± 0.009	0.028 ± 0.005	0.032 ± 0.023	0.028 ± 0.007	0.026 ± 0.008

A satellite group was given the vehicle control or Thai rice instant granules containing turmeric extract and *Phyllanthus emblica* fruit pulp at 2.000 mg/kg daily for 180 days followed by no treatment for 28 days.

Values are expressed as mean ± SD.

^a^ Significantly different from control, *p* < 0.05.

#### 3.3.2 Hematological and biochemical parameters

The effect of TR instant granules on hematological parameters is presented in Table 3. No significant changes were found in the hematological parameters in male and female rats treated with TR instant granules. There were no significant changes in any parameters in the satellite male and female rats treated with TR instant granules.

**TABLE 3:** Hematological parameters of Wistar rats receiving Thai rice instant granules containing turmeric extract and *Phyllanthus emblica* fruit pulp in the chronic toxicity test.

Hematological parameters	Control	Main group			Satellite group	
		200 mg/kg	600 mg/kg	2000 mg/kg	Control	2000 mg/kg
Males						
RBC (×10^6^/μL)	8.98 ± 0.50	9.19 ± 0.38	8.84 ± 0.23	8.90 ± 0.31	8.93 ± 0.50	8.76 ± 0.23
Hb (g/dL)	15.85 ± 0.65	16.34 ± 0.53	16.01 ± 0.36	16.31 ± 0.43	15.88 ± 0.46	15.94 ± 0.55
HCT (%)	49.19 ± 2.37	49.85 ± 1.65	48.56 ± 1.36	49.71 ± 1.58	47.35 ± 2.87	47.46 ± 1.98
MCV (fL)	54.81 ± 1.73	54.27 ± 1.49	54.93 ± 0.61	55.88 ± 1.35	53.10 ± 1.66	54.16 ± 2.12
MCH (pg)	17.67 ± 0.44	17.79 ± 0.60	18.10 ± 0.31	18.52 ± 0.52	17.80 ± 0.88	18.16 ± 0.59
MCHC (g/dL)	32.26 ± 0.49	32.78 ± 0.30	32.98 ± 0.30	32.83 ± 0.49	33.60 ± 1.47	33.58 ± 0.54
PLT (×10^3^ cell/μL)	1026 ± 89	963 ± 61	938 ± 132	949 ± 75	1065 ± 158	933 ± 74
WBC (×10^3^ cell/μL)	6.09 ± 0.84	6.42 ± 1.29	5.52 ± 1.82	6.41 ± 1.28	5.87 ± 0.96	4.78 ± 1.31
Neu (%)	13.56 ± 3.33	14.96 ± 2.49	18.61 ± 8.51	23.12 ± 3.11	18.08 ± 2.57	18.86 ± 5.09
Lym (%)	79.11 ± 3.76	77.51 ± 3.77	72.33 ± 9.21	67.11 ± 14.94	75.10 ± 2.44	74.32 ± 4.58
Mon (%)	6.21 ± 2.59	6.35 ± 1.65	8.02 ± 2.88	8.52 ± 2.99	5.00 ± 0.68	5.28 ± 1.43
Eos (%)	1.11 ± 0.24	1.17 ± 0.25	1.02 ± 0.44	1.23 ± 0.40	1.83 ± 0.64	1.54 ± 0.64
Bas (%)	0.01 ± 0.03	0.01 ± 0.03	0.01 ± 0.03	0.01 ± 0.03	0.00 ± 0.00	0.00 ± 0.00
Females						
RBC (×10^6^/μL)	8.10 ± 0.30	7.69 ± 0.41	7.79 ± 0.31	7.74 ± 0.41	7.72 ± 0.20	7.57 ± 0.40
Hb (g/dL)	15.90 ± 0.45	15.22 ± 0.48	15.34 ± 0.95	15.38 ± 0.64	15.60 ± 0.47	14.94 ± 0.59
HCT (%)	47.00 ± 1.82	45.52 ± 1.41	45.09 ± 2.53	45.78 ± 1.98	45.92 ± 1.49	44.28 ± 1.98
MCV (fL)	58.06 ± 1.61	59.23 ± 1.89	57.88 ± 1.95	59.23 ± 1.51	59.50 ± 0.75	58.58 ± 1.74
MCH (pg)	19.65 ± 0.40	19.80 ± 0.65	19.70 ± 0.64	19.90 ± 0.54	20.24 ± 0.37	19.76 ± 0.61
MCHC (g/dL)	33.85 ± 0.51	33.43 ± 0.38	34.02 ± 0.48	33.61 ± 0.38	33.96 ± 0.35	33.74 ± 0.21
PLT (×10^3^ cell/μL)	976 ± 150	1003 ± 142	996 ± 96	1036 ± 134	1018 ± 164	908 ± 130
WBC (×10^3^ cell/μL)	4.36 ± 1.09	4.33 ± 0.85	4.07 ± 0.87	3.89 ± 1.22	4.63 ± 1.14	3.98 ± 0.63
Neu (%)	11.12 ± 3.10	11.40 ± 1.27	16.58 ± 3.70	17.24 ± 8.38	10.80 ± 1.65	16.44 ± 8.46
Lym (%)	83.24 ± 4.31	80.65 ± 3.86	76.62 ± 5.41	75.89 ± 9.91	85.14 ± 1.01	76.58 ± 11.62
Mon (%)	4.37 ± 1.84	6.91 ± 3.43	5.32 ± 2.65	4.83 ± 3.81	3.12 ± 1.31	5.22 ± 3.09
Eos (%)	1.27 ± 0.51	1.04 ± 0.31	1.48 ± 0.71	1.41 ± 0.60	0.92 ± 0.34	1.76 ± 0.83
Bas (%)	0.00 ± 0.00	0.00 ± 0.00	0.00 ± 0.00	0.63 ± 1.99	0.02 ± 0.04	0.00 ± 0.00

A satellite group was given the vehicle control or Thai rice instant granules containing turmeric extract and *Phyllanthus emblica* fruit pulp at 2,000 mg/kg BW, daily for 180 days followed by no treatment for 28 days. RBC: red blood cell count, Hb: hemoglobin, HCT: hematocrit, MCV: mean corpuscular volume, MCH: mean corpuscular hemoglobin, MCHC: mean corpuscular hemoglobin concentration, PLT: platelet count, WBC: white blood cell count, Neu: neutrophil, Lym: lymphocyte, Mon: monocyte, Eos: eosinophil, Bas: basophil. Values are expressed as mean ± SD., No significant differences were found between the treatment groups and the control group (*P* ≥ 0.05).

Clinical blood chemistry parameters of rats treated with TR instant granules are shown in Table 4. TR instant granules (200 mg/kg BW) caused a significant increase in the levels of total protein and albumin in male rats. A significant decrease in total protein levels was observed in female rats at doses of 600 and 2,000 mg/kg BW. The levels of albumin, AST, and ALT were significantly decreased in rats treated with TR instant granules (2,000 mg/kg BW). In the satellite group, albumin and AST levels were significantly increased when compared to the control group. There was no significant difference in other parameters.

**TABLE 4:** Biochemical parameters of rats receiving Thai rice instant-granules containing turmeric extract and *Phyllanthus emblica* fruit pulp in the chronic toxicity test.

Biochemical parameters	Control	Main group			Satellite group	
		200 mg/kg	600 mg/kg	2000 mg/kg	Control	2000 mg/kg
Males						
Cholesterol (mg/dL)	91.78 ± 13.49	83.40 ± 14.45	80.22 ± 15.72	85.56 ± 20.60	94.25 ± 29.07	83.60 ± 8.08
BUN (mg/dL)	16.50 ± 1.29	17.52 ± 1.35	15.66 ± 1.23	16.23 ± 1.05	16.95 ± 2.50	16.02 ± 1.43
Creatinine (mg/dL)	0.70 ± 0.08	0.71 ± 0.05	0.69 ± 0.08	0.69 ± 0.07	0.72 ± 0.12	0.72 ± 0.07
Total protein (g/dL)	7.33 ± 0.35	7.66 ± 0.22*	7.22 ± 0.33	7.34 ± 0.17	6.88 ± 0.35	6.82 ± 0.28
Albumin (g/dL)	3.47 ± 0.15	3.71 ± 0.12*	3.53 ± 0.17	3.47 ± 0.10	3.45 ± 0.17	3.48 ± 0.11
AST (U/L)	143.43 ± 46.98	198.00 ± 48.68	135.29 ± 22.29	178.71 ± 38.95	91.33 ± 26.50	98.25 ± 12.54
ALT (U/L)	64.89 ± 25.73	100.63 ± 38.87	83.25 ± 27.76	103.33 ± 42.15	40.00 ± 4.58	50.50 ± 8.70
ALP (U/L)	64.78 ± 5.95	65.75 ± 8.26	61.75 ± 7.34	59.71 ± 5.94	46.00 ± 3.16	50.80 ± 8.98
Total bilirubin (mg/dL)	0.11 ± 0.02	0.10 ± 0.02	0.10 ± 0.01	0.11 ± 0.02	0.16 ± 0.02	0.17 ± 0.01
Direct bilirubin (mg/dL)	0.05 ± 0.01	0.05 ± 0.01	0.05 ± 0.01	0.05 ± 0.01	0.10 ± 0.01	0.10 ± 0.01
Na^+^ (mmol/L)	145.03 ± 1.69	146.00 ± 1.35	144.51 ± 1.57	145.19 ± 1.34	144.60 ± 1.94	144.28 ± 1.31
K^+^ (mmol/L)	6.30 ± 1.15	6.72 ± 0.98	6.35 ± 0.78	6.70 ± 1.14	6.55 ± 2.56	6.69 ± 1.43
Cl^−^ (mmol/L)	96.36 ± 1.95	96.76 ± 1.15	95.86 ± 1.90	95.32 ± 1.56	97.38 ± 2.93	99.64 ± 1.19
TCO_2_ (mmol/L)	31.70 ± 1.38	31.85 ± 2.21	30.61 ± 1.92	31.86 ± 1.51	34.73 ± 2.10	34.74 ± 1.54
Females						
Cholesterol (mg/dL)	86.80 ± 15.33	82.30 ± 22.57	78.80 ± 19.96	66.60 ± 22.19	74.40 ± 6.35	77.20 ± 17.88
BUN (mg/dL)	20.26 ± 2.16	18.36 ± 2.38	18.84 ± 2.43	18.81 ± 2.89	17.96 ± 2.11	18.84 ± 2.35
Creatinine (mg/dL)	0.82 ± 0.07	0.86 ± 0.06	0.83 ± 0.05	0.84 ± 0.07	0.81 ± 0.03	0.84 ± 0.04
Total protein (g/dL)	9.59 ± 0.41	9.41 ± 0.37	9.01 ± 0.42^a^	8.81 ± 0.78^a^	7.82 ± 0.46	7.78 ± 0.15
Albumin (g/dL)	4.60 ± 0.19	4.60 ± 0.20	4.44 ± 0.16	4.32 ± 0.33^a^	4.16 ± 0.11	4.34 ± 0.11^a^
AST (U/L)	176.00 ± 38.75	151.22 ± 29.25	160.13 ± 78.20	93.33 ± 17.73^a^	86.75 ± 6.18	168.75 ± 63.25^a^
ALT (U/L)	57.88 ± 14.81	57.88 ± 8.87	77.67 ± 44.14	39.57 ± 4.31^a^	46.00 ± 8.37	67.75 ± 18.00
ALP (U/L)	18.57 ± 2.23	19.86 ± 1.35	20.56 ± 3.13	19.17 ± 2.64	17.50 ± 4.04	18.00 ± 3.08
Total bilirubin (mg/dL)	0.16 ± 0.02	0.15 ± 0.03	0.16 ± 0.03	0.15 ± 0.04	0.22 ± 0.03	0.22 ± 0.02
Direct bilirubin (mg/dL)	0.08 ± 0.02	0.07 ± 0.02	0.08 ± 0.02	0.08 ± 0.02	0.13 ± 0.02	0.13 ± 0.02
Na^+^ (mmol/L)	144.77 ± 2.78	144.35 ± 2.02	144.32 ± 4.58	141.57 ± 3.81	143.54 ± 1.90	144.90 ± 0.45
K^+^ (mmol/L)	5.62 ± 0.34	6.33 ± 1.65	5.93 ± 1.01	6.40 ± 1.82	6.77 ± 1.55	7.02 ± 1.52
Cl^−^ (mmol/L)	98.04 ± 1.66	90.58 ± 23.14	97.80 ± 2.37	97.13 ± 1.65	98.08 ± 1.32	98.26 ± 1.34
TCO_2_ (mmol/L)	27.55 ± 1.64	26.42 ± 2.03	27.58 ± 2.14	26.09 ± 2.01	28.82 ± 1.61	27.96 ± 2.48

A satellite group was given the vehicle control or Thai rice instant granules containing turmeric extract and *Phyllanthus emblica* fruit pulp at 2000 mg/kg BW, daily for 180 days followed by no treatment for 28 days. Values are expressed as mean ± SD.

^a^ Significantly different from control, *p* < 0.05.

#### 3.3.3 Histopathological examination

**FIGURE 3:**
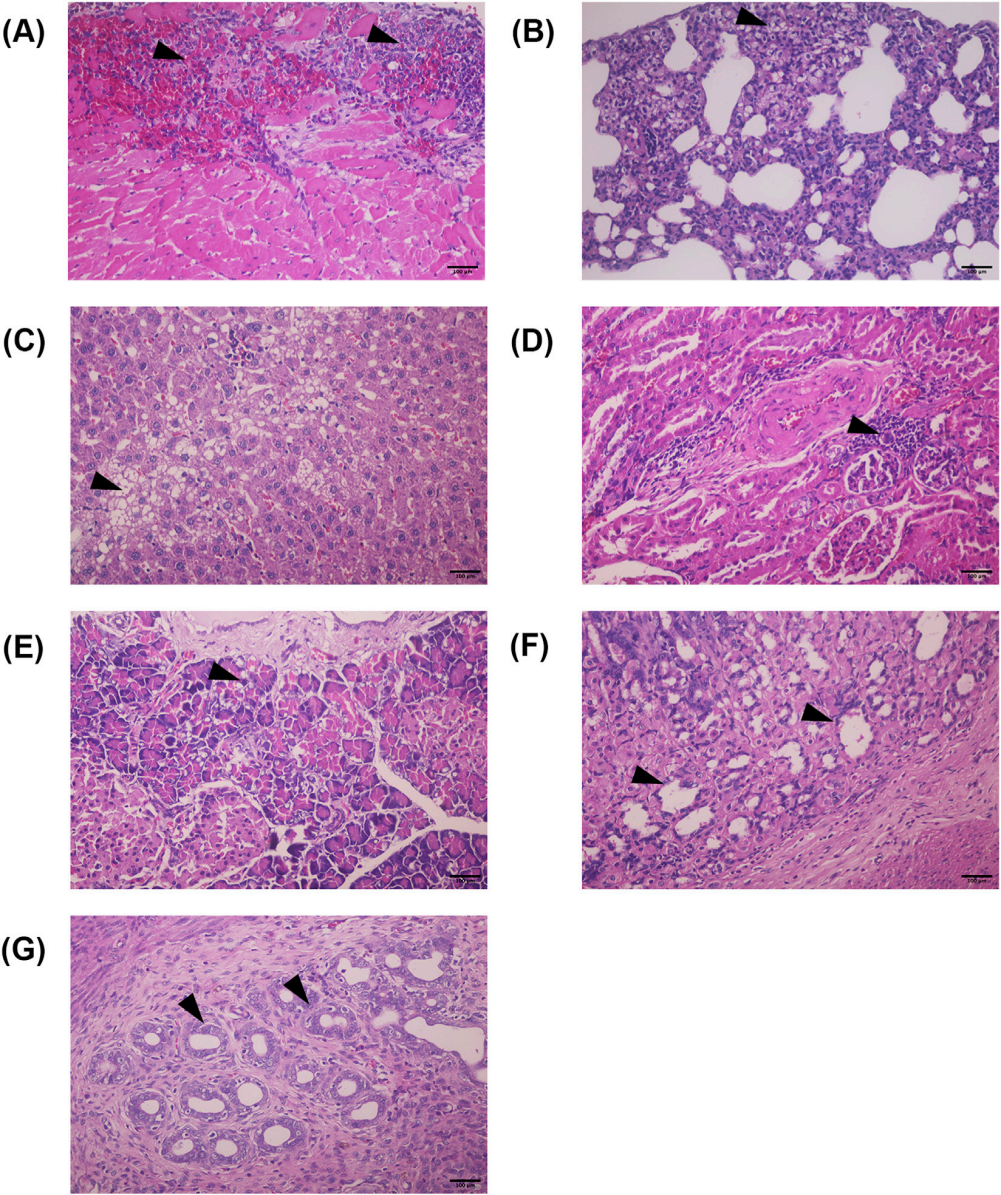
Photomicrographs of histological findings of different organs from the chronic toxicity study of TR instant granules (H&E staining, 40 × magnification). **(A)** Myocarditis (Heart), **(B)** Foamy macrophage infiltration (Lung), **(C)** Fatty degeneration (Liver), **(D)** Interstitial nephritis (Kidney), **(E)** Pancreatic duct hyperplasia/tumor pancreas (Pancreas), **(F)** Dilated gland/glandular cyst (Stomach), **(G)** Hyperplasia (Uterine gland). Abnormal lesions are indicated with arrowheads. HE. 400x. Scale bar = 100 μm.

Histopathological findings for male and female rats are summarized in Supplementary Table S3. In this study, histopathological changes were assessed in rats treated with a high dose (2,000 mg/kg BW) of TR instant granules, comparing both main and satellite groups to controls. Representative photomicrographs of histological findings from various organs in the chronic toxicity study of TR instant granules are shown in Figure 3. At 2,000 mg/kg BW, no abnormalities were observed in the brain or lungs of either male or female rats. Myocarditis was detected in male rats (main group: 2/10; satellite group: 1/5). Fatty degeneration of the liver occurred in both males (7/10) and females (3/10) in the main group, as well as in satellite males (2/5) and females (1/5). Bile duct proliferation was seen in one female (1/10), and hepatitis/cholangiohepatitis was only observed in the female satellite group (1/5). Renal changes included tubular cast (female, 1/10) and interstitial nephritis (male and female, 1/10 each). Pancreatitis (1/5, male) and pancreatic duct hyperplasia/tumor (1/5, female) were found in the satellite group. No histopathological changes were found in the spleen, thymus, stomach, adrenal glands, or reproductive organs of male and female rats receiving TR instant granules.

#### 3.3.4 Effect of TR instant granules on the expression of antioxidant genes

**FIGURE 4:**
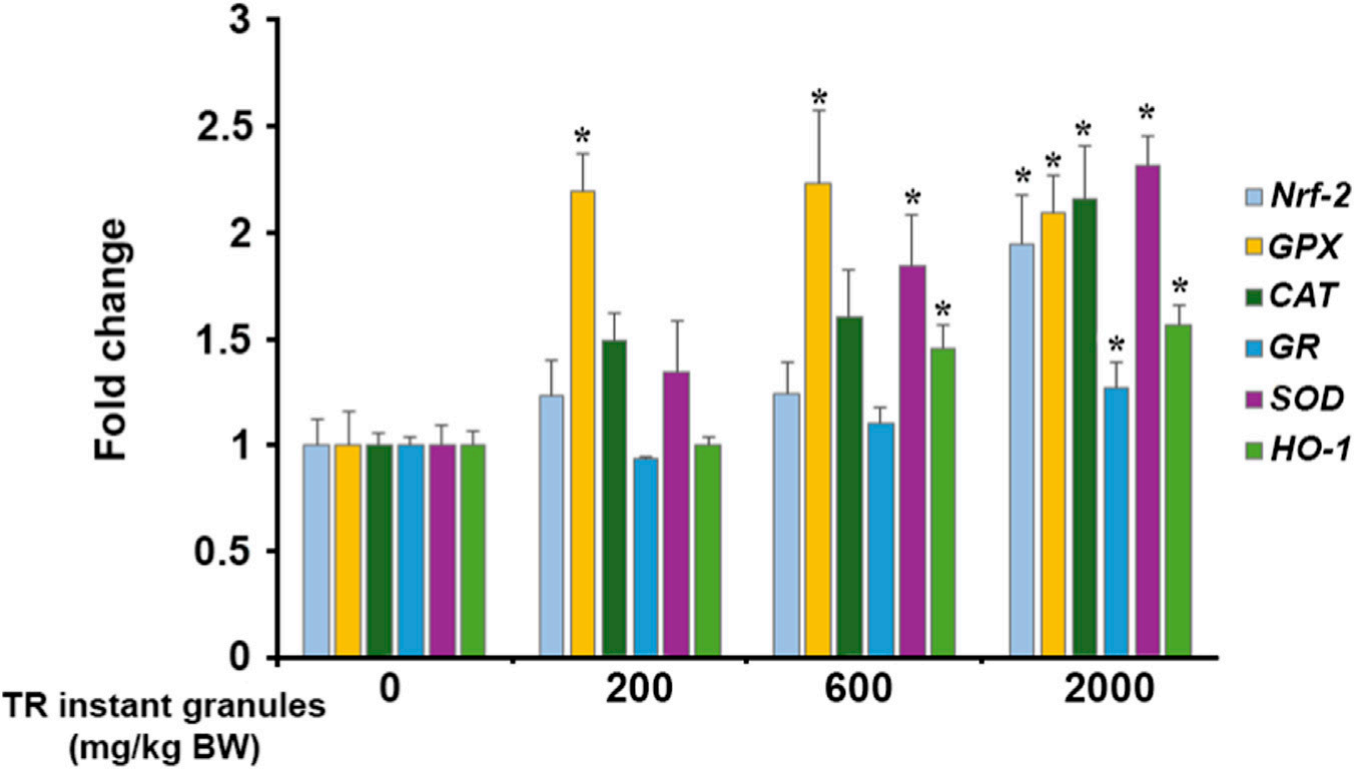
Effect of TR instant granules on antioxidant gene expression in male rats. Values are expressed as mean ± SEM. Nrf2: nuclear factor erythroid-2 related factor 2, GPX: glutathione peroxidase, CAT: catalase, GR: glutathione reductase, SOD: superoxide dismutase, HO-1: heme oxygenase, TR instant granules: Thai rice instant granules containing turmeric extract and *Phyllanthus emblica* fruit pulp, BW: body weight. *Significantly different from control group, *p* < 0.05.

To assess the long-term effects of TR instant granules on antioxidant status, the expression of antioxidant genes was evaluated. It was found that treatment with 2,000 mg/kg BW of TR instant granules for 180 days significantly upregulated the expression of nuclear factor erythroid-2 related factor 2 (*Nrf2*), glutathione peroxidase (*GPX*), catalase (*CAT*), glutathione reductase (*GR*), superoxide dismutase (*SOD*), and heme oxygenase (*H O -1*). Treatment with 600 mg/kg BW of TR instant granules showed a significant effect on the mRNA levels of *GPX, SOD,* and *H O -1*, whereas 200 mg/kg BW of TR instant granules significantly induced only the *GPX* gene (Figure 4).

### 3.4 In silico study

**FIGURE 5:**
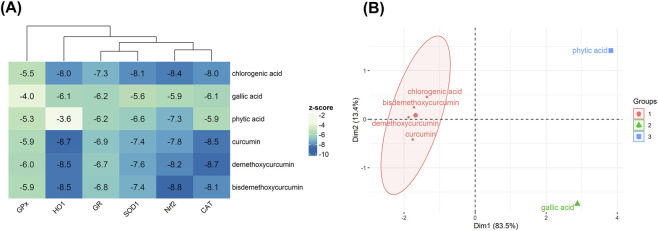
Molecular docking analysis of phytochemicals present in TR instant granules with antioxidant targets. **(A)** A Heatmap of molecular docking results (kcal/mol) for six phytochemicals against antioxidant enzymes and the Nrf2–Keap1 complex, accompanied by hierarchical clustering of both ligands and protein targets. **(B)** Principal component analysis (PCA) plot illustrating the grouping of phytochemicals into three clusters based on their binding affinity profiles.

The molecular docking analysis illustrated the various binding affinity patterns of the six phytochemicals in TR instant granules on the selected antioxidant enzymes (GPx, HO-1, GR, SOD1, CAT) and the Nrf2–Keap1 complex (Figure 5). Of all the compounds, chlorogenic acid exhibited the strongest overall binding profile with particularly high affinities for Nrf2 (−8.4 kcal/mol), CAT (−8.0 kcal/mol), HO-1 (−8.0 kcal/mol), and SOD1 (−8.1 kcal/mol). Curcumin and its derivatives exhibited consistently high binding affinities in molecular docking studies. Specifically, demethoxycurcumin showed notable interactions with HO-1 (−8.5 kcal/mol), CAT (−8.7 kcal/mol), and Nrf2 (−8.2 kcal/mol). Among the tested compounds, bisdemethoxycurcumin demonstrated the highest affinity for Nrf2 (−8.8 kcal/mol). In contrast, gallic acid and phytic acid generally exhibited weaker binding, with the lowest affinities observed for HO-1 (−6.1 kcal/mol) and GR (−3.6 kcal/mol), respectively. The binding affinities of GPx were moderate across all ligands, ranging from −4.0 kcal/mol for gallic acid to −6.0 kcal/mol for demethoxycurcumin. The hierarchical clustering pattern in the heatmap showed that the curcuminoids clustered together due to their strong and broad-spectrum binding across multiple antioxidant targets, whereas smaller phenolic acids, such as gallic acid and phytic acid, formed a separate cluster reflecting their comparatively weaker affinities (Figure 5A). These findings indicate that chlorogenic acid, curcumin, and its derivatives have the greatest potential for multi-target antioxidant regulation through strong interactions with key enzymes and Nrf2.

Principal component analysis (PCA) differentiated the phytochemicals into three major groups based on their binding profiles (Figure 5B). The first principal component (Dim1, 83.5%) accounted for most of the variance and revealed a clear separation between the groups. Chlorogenic acid, curcumin, demethoxycurcumin, and bisdemethoxycurcumin clustered closely, reflecting their similar broad-spectrum and high-affinity binding patterns. Gallic acid formed a distinct cluster, characterized by moderate binding across most targets, while phytic acid was separated due to its markedly lower binding affinities, particularly towards HO-1. These findings suggest that polyphenolic compounds, especially chlorogenic acid and curcuminoids, have strong multi-target antioxidant potential through stable interactions with both enzymatic and transcription factor-associated antioxidant systems, while smaller phenolic acids and phytic acid exhibit more limited binding activity.

**FIGURE 6:**
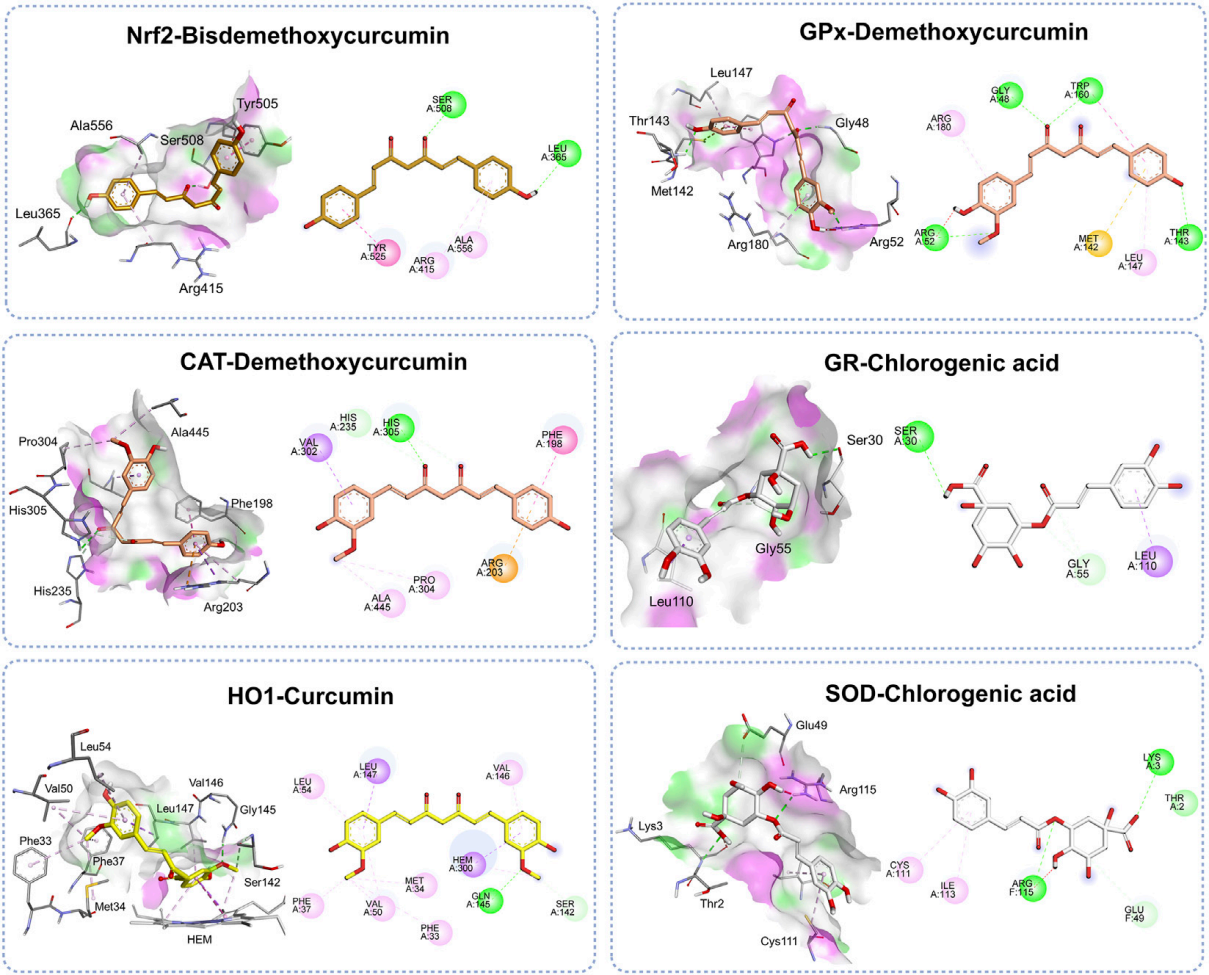
Molecular binding interactions between phytochemicals from TR instant granules and antioxidant target proteins. Representative three-dimensional (left) and two-dimensional (right) binding poses with key interactions for the top compound–target complexes. The surface color gradient represents hydrogen-bond donors (pink) and acceptors (green). Green dashed lines illustrate hydrogen bonds, and pink or purple dashed lines indicate hydrophobic interactions.

Figure 6 shows the molecular binding patterns of phytochemicals from TR instant granules and target proteins. The docking poses and amino acid contacts for each complex were examined. Bisdemethoxycurcumin showed the highest binding affinity towards Nrf2 by targeting the Kelch domain of Keap1, which accommodates the Neh2 domain of Nrf2 (Lo et al., 2006). It formed hydrogen bonds with Leu365 and Ser508, along with hydrophobic interactions involving Arg415, Ala556, and Tyr525, residues corresponding to the key pocket identified in structural analyses (e.g., Arg415 and Ser508 in P1; Ala556 in P3; Tyr525 in P4) (Canning et al., 2015). These interactions are critical for molecular recognition specificity and contribute substantially to the stability of the ligand–protein complex (Lo et al., 2006; Bello and Morales-González, 2017; Ma et al., 2020). These findings provide a structural rationale for the high binding specificity of bisdemethoxycurcumin and support docking evidence suggesting that it may competitively bind Keap1, thereby promoting Nrf2 activation.

Demethoxycurcumin bound favorably within the binding site of glutathione peroxidase 1 (GPx1), forming hydrogen bonds with Gly48, Arg52, Thr143, and Trp160 that anchored the ligand within the pocket. Additional stabilization was provided by a *π*–*π* interaction with Trp160, *π*–alkyl interactions with Leu147 and Arg180, and a *π*–sulfur contact with Met142. Several of these residues are located in or near the substrate-binding region and are involved in ligand positioning and stabilization, thereby facilitating the reduction of hydrogen peroxide and organic hydroperoxides using reduced glutathione as an electron donor (Toppo et al., 2009). Given the role of GPx1 in maintaining cellular redox homeostasis by preventing the accumulation of harmful peroxides, this interaction profile supports the potential of demethoxycurcumin as a non-covalent modulator of GPx1 activity.

In catalase (CAT), demethoxycurcumin bound favorably within the NADPH binding site, forming hydrogen bonds and hydrophobic interactions that stabilized the ligand (Putnam et al., 2000). Specifically, hydrogen bonds were formed with His305 and His235, while additional stabilization arose from hydrophobic interactions involving Phe198, Arg203, Val302, Ala445, and Pro304. These residues are located within the substrate access channel and surrounding hydrophobic pocket, suggesting that demethoxycurcumin can occupy key regions near the catalytic center, potentially influencing substrate accessibility and modulating enzyme activity.

For glutathione reductase (GR), chlorogenic acid was accommodated within the binding site corresponding to the binding site of the ajoene inhibitor (Gallwitz et al., 1999), and stabilized through hydrogen bonding and hydrophobic interactions. Chlorogenic acid formed hydrogen bonds with Ser30 and Gly55, located near the FAD-binding region, which is essential for catalyzing the reduction of oxidized glutathione (GSSG) to its reduced form (GSH) (Berkholz et al., 2008). Additional *π*–sigma interaction with Leu110 further enhanced the complex stability. The combination of polar and hydrophobic contacts indicates that chlorogenic acid engages residues critically for substrate positioning and electron transfer, potentially modulating catalytic efficiency.

Curcumin bound strongly within the active site of heme oxygenase-1 (HO-1) and engaged in a network of stabilizing interactions. These may contribute to its potential as an HO-1 modulator. Hydrogen bonds were formed with Gln145 and Ser142, positioning the molecule near the catalytic heme cofactor. Additional stabilization was observed through multiple hydrophobic interactions with Leu54, Val50, Val146, Leu147, Met34, Phe33, Phe37, as well as *π*–sigma interaction with the heme group. These contacts are consistent with the structural organization of the HO-1 catalytic pocket, in which hydrophobic residues anchor ligands in proximity to the heme iron to facilitate modulation of enzymatic activity (Rahman et al., 2013).

For superoxide dismutase (SOD), chlorogenic acid bound within the binding-site region and formed multiple stabilizing interactions. These may contribute to its modulatory potential. Hydrogen bonds were established with Lys3, Thr2, Glu49, and Arg115. Additional stabilization was provided by a *π*–alkyl interaction with Cys111 and Ile113, contributing to hydrophobic interactions. Several of these residues are located near the catalytic metal-binding site, which is essential for the dismutation of superoxide radicals into oxygen and hydrogen peroxide (Strange et al., 2006). Chlorogenic acid may enhance antioxidant activity by interacting with residues near this catalytic region and affecting substrate accessibility or enzyme conformational dynamics.

## 4 Discussion

Alternative medicines derived from natural products and edible plants, which have an acceptable safety profile, may become more popular in the pharmaceutical market. Although the global demand for herbal medicine has increased, there are still concerns about its use and safety (Ezenyi et al., 2013; Elkordy et al., 2021). A chronic repeated–dose toxicity study is a long-term study conducted to evaluate the potential harmful effects of chemicals or substances required to support long-term dosing in clinical trials (Prior et al., 2024). Many plants produce toxic secondary metabolites, as documented in previous reports (Ezenyi et al., 2013). Therefore, it is still important to assess the toxicological properties of any herbal medicine.

Recently, our group has produced Thai rice instant granules containing turmeric extract and *P. emblica* fruit pulp, using the granulation method to enhance the solubility and absorption of the active substance. Despite extensive research on the pharmacological and toxicological properties of turmeric extract and *P. emblica* fruit (Qureshi et al., 1992; Jaijoy et al., 2011; Soleimani et al., 2018), it is crucial to assess their toxicity profiles, as this is a new product development. To ensure the safety of this product, animal toxicity tests are required to identify potential adverse effects and safety dosages before the product is used in clinical trials on humans.

In the present study, we evaluated the chronic oral toxicity of the TR instant granules in Wistar rats over 180 days. Both male and female rats were assigned to three dosage groups (200, 600, and 2,000 mg/kg BW), and none showed signs of toxicity or mortality. The treated groups exhibited no differences in body weight, food intake, or water consumption compared to the control group, suggesting no impact on overall animal health. Changes in organ weight are frequently linked to treatment-related toxic effects (Sellers et al., 2007; Lazic et al., 2020). We found that 2,000 mg/kg BW of TR instant granules increased the relative liver weight in both male and female rats compared to the control group. Microscopic and clinical pathological examinations showed that TR instant granules caused significant fatty degeneration in rats (7/10 in males and 3/10 in females). However, these abnormalities were reduced in the satellite group that received 2,000 mg/kg BW of TR instant granules. These findings revealed that TR instant granules have hepatotoxic effects that depend on the duration of consumption. Although the relative pancreas and lung weights were also increased in male rats treated with TR instant granules, the Society of Toxicologic Pathology (STP) considers lung weights to be a standard endpoint for test compounds in inhalation studies. Additionally, the pancreas is difficult to identify in rodents because it is interspersed with adipose tissue and lymph nodes (Sellers et al., 2007). The toxic effects caused by the test product on the lungs and spleen were confirmed through clinical pathology and microscopic findings. The results showed that no histopathological changes were observed in rats treated with TR instant granules. These results indicate that TR instant granules did not have any adverse effects on the lung or spleen.

Hematological and biochemical measurements provide important information that relates to organs and tissues in toxicology and safety studies (Patel et al., 2024). Tissue damage caused by toxic substances can result in direct cell destruction, which may lead to biochemical and hematological changes (Arika et al., 2016). The hematological examination indicated that there was no significant difference between the groups treated with TR instant granules and the control group in all parameters. Similar results were also observed in the satellite group. However, the levels of PLT in male and female rats in the main and satellite groups were above the normal range (normal ranges: 412.25–849.25 10^3^/μL in males and 377.63–963.83 10^3^/μL in females), as well as the levels of Hb in male rats (normal range: 12.8–15.80 in males) (Patel et al., 2024). It has been found that hematology results are linked to the duration of fasting. Increased fasting duration led to an increase in Hb levels in male rats and high platelet levels in both male and female rats (Kale et al., 2009). In the present study, blood collection was performed after 16 h of overnight fasting. The increase in hemoglobin and platelet levels may be associated with the duration of fasting. Based on these observations, TR instant granules can be safely used in rats at a dose of up to 2,000 mg/kg BW without any negative effects on all hematological parameters.

The measurement of biochemical parameters provides information about the adverse effects of a chemical substance on the functional status of major organ systems, such as the liver and kidneys (Dzoyem et al., 2014). In the present study, a clinical blood chemistry analysis was conducted to quantify any toxic effects on liver function (AST, ALT, ALP, total protein, albumin, and bilirubin) and renal function (BUN and creatinine). Moreover, total cholesterol, electrolytes (Na^+^, K^+^, Cl^−^), and total carbon dioxide content (TCO_2_) were also evaluated. The treatment with TR instant granules had a significant impact on blood chemistry related to liver function, which includes AST, ALT, total protein, and albumin. It is well known that changes in the level of these indicators indicate abnormalities in liver cell function or damage (Intatham et al., 2024). Albumin is a significant protein that constitutes 50%–60% of the total protein in serum. It is synthesized in the liver and is considered a marker for the liver’s synthetic function (Kalas et al., 2021; Thakur et al., 2024). Although the albumin levels were high in rats receiving TR instant granules, all these values remained within normal ranges (3.48–4.9 g/dL in males and 3.58–5.4 g/dL in females (Patel et al., 2024). The total protein level in rats administered TR instant granules and the control group was above the normal ranges (6.07–7.4 g/dL in males and 6.16–7.83 g/dL in females) (Patel et al., 2024). After withdrawal, the increase in albumin and total protein levels diminished and reached the normal range. However, although TR instant granules significantly induced an increase in AST and ALT levels in female rats, all these values remained within normal ranges. These results demonstrate that long-term treatment with TR instant granules could affect liver tissue, as supported by the relative liver weight and histopathological analysis. Even though other serum parameters were elevated in the groups treated with TR instant granules, they reverted to their normal ranges after withdrawal.

Histopathological examinations of internal organs provide information about the abnormalities in the organ caused by the test substance (Schafer et al., 2018). The present study showed no significant pathological changes between the group treated with TR instant granules and the control group. The satellite group showed similar results. Although TR instant granules caused high liver fatty degeneration in rats, the abnormalities were reduced after 28 days of withdrawal. Furthermore, the tubular cast in the kidney was not found after withdrawal. According to these findings, the administration of TR instant granules in doses up to 2,000 mg/kg BW for 180 days has no adverse effect on the analyzed organs.

Although no chronic toxic effects of turmeric extract have been reported, a previous subchronic toxicity study showed that the highest tested dose of 200 mg/kg caused no toxic effects in rats (Mulyani et al., 2022). In the present study, the TR instant granules contain 20% turmeric extract; therefore, a dose of 2,000 mg/kg of TR instant granules contains approximately 400 mg/kg of turmeric extract, which is twice the maximum dose evaluated in previous studies. These findings support the long-term safety data of turmeric extract. Furthermore, based on our findings, a dose of 2,000 mg/kg body weight in rats corresponds to an approximate human equivalent dose of 20,000 mg/60 kg (Nair and Jacob, 2016). As a result, TR instant granules were considered safe in the chronic toxicity test.

Polyphenols are well recognized as potent natural antioxidants that protect cellular components from oxidative damage. They exert their antioxidant effects through various mechanisms, including free radical scavenging, metal ion chelation, and modulation of antioxidant enzyme activities (Chibuye et al., 2024; Hano and Tungmunnithum, 2020; Tumilaar et al., 2024; Gan et al., 2025). In the present study, the antioxidant effects of TR instant granules are primarily attributed to their major phytochemicals, chlorogenic acid and curcuminoids, as revealed by phytochemical analysis. Chlorogenic acid and curcumin have been well documented for their ability to enhance antioxidant enzymes and activate the Nrf2 pathway (Nguyen et al., 2024; Kim et al., 2025). An *in silico* study further demonstrated that chlorogenic acid and curcuminoids have strong binding affinities to several key antioxidant enzymes, including GPx1, CAT, GR, SOD1, HO-1, as well as the Nrf2–Keap1 complex. This computational technique, which is critical for predicting small molecule–protein interactions and elucidating antioxidant mechanisms (Meng et al., 2011; Srivastava et al., 2023), supports the findings from animal studies in which TR instant granules were shown to upregulate the expression of antioxidant genes in the liver. The results suggest that TR instant granules enhance liver antioxidant defenses by directly interacting with the key antioxidant pathway and enzymes. In addition to these major phytochemicals, other phenolic compounds present at lower concentrations, such as phytic acid, gallic acid, quercetin, rosmarinic acid, epicatechin, and caffeic acid, may also contribute synergistically to the overall antioxidant capacity of the TR instant granules.

Our study demonstrated that TR instant granules exhibited no mortality or significant adverse effects in rats during a 180-day administration period at doses up to 2,000 mg/kg body weight. Although some biochemical parameters related to liver function (total protein, albumin, AST, and ALT) and minor histopathological changes were observed, these alterations returned to normal following withdrawal of the granules. Consequently, the No Observed Adverse Effect Level (NOAEL) was determined to be 2,000 mg/kg/day for both male and female rats. Additionally, long-term administration of TR instant granules significantly upregulated the expression of antioxidant enzyme genes in the liver, and *in silico* studies confirmed strong binding interactions between the bioactive compounds and key antioxidant proteins. The safety profile and demonstrated antioxidant properties found in this study support the potential for long-term use of TR instant granules in clinical trials. However, it is important to note that this study did not include a pharmacokinetic evaluation of TR instant granules, and further research is needed to understand their metabolism and systemic effects.

To provide a more balanced and convincing mechanistic foundation, more comprehensive and well-controlled antioxidant validation experiments should be conducted. Clinical trials in humans are necessary to confirm the safety and effectiveness of TR instant granules for clinical use.

## Data Availability

The original contributions presented in the study are included in the article/Supplementary Material, further inquiries can be directed to the corresponding author.
