# Supplementary material for: Thai rice instant granules containing turmeric extract and Phyllanthus emblica fruit pulp: Chronic toxicity and antioxidant profiles in rats and in silico investigation of bioactive compounds
Source: Front Toxicol. 2025 Dec 5;7:1691014. doi: 10.3389/ftox.2025.1691014 (PMC12714627; doi:10.3389/ftox.2025.1691014)
Supplement: Supplementary file 1 [file Table1.docx]

Supplementary Material

**Table S1** Primer sequences used for RT-PCR

| **Gene** | **5′-3′ Primer sequence** |
| --- | --- |
| *Nrf-2* | Forward: 5'-GCC AGC TGA ACT CCT TAG AC-3'  Reverse: 5'-GAT TCG TGC ACA GCA GCA-3' |
| *GPX* | Forward: 5′-CTC TCC GCG GTG GCA CAG T-3'  Reverse: 5′-CCA CCA CCG GGT CGG ACA TAC-3' |
| *CAT* | Forward: 5′-GCG AAT GGA GAG GCA GTG TAC-3'  Reverse: 5′-GAG TGA CGT TGT CTT CAT TAG CAC TG-3' |
| *GR* | Forward: 5’-TGA GCC GCC TGA ACA ACA-3’  Reverse: 5’-TTG CGT AGC CGT GGA TGA C-3’ |
| *Cu-Zn SOD* | Forward: 5-GCA GAA GGC AAG CGG TGA AC-3'  Reverse: 5-TAG CAG GAC AGC AGA TGA GT-3' |
| *HO-1* | Forward: 5'-ACA GGG TGA CAG AAG AGG CTA A-3'  Reverse: 5'-CTG TGA GGG ACT CTG GTC TTT G-3' |
| *β-Actin* | Forward: 5'-ACA GGA TGC AGA AGG AGA TTA C-3'  Reverse: 5'-AGA GTG AGG CCA GGA TAG A-3' |

**Table S2** General observations from a 180-day oral toxicity study of Thai rice instant-granules containing turmeric extract and *Phyllanthus emblica* fruit pulp in rats

| **Sex** | **Group** | **TR instant-granules**  **(mg/kg BW)** | **Body weight (g)** | | **Food consumption**  **(g/rat/day)** | **Water intake**  **(ml/rat/day)** |
| --- | --- | --- | --- | --- | --- | --- |
|  |  |  | **Initial weight** | **Final weight** |  |  |
| **Males** |  | Control | 158 ± 12 | 329 ± 29 | 15.3 ± 2.2 | 23.3 ± 2.6 |
|  | Treatment | 200 | 159 ± 10 | 321 ± 36 | 14.7 ± 1.4 | 23.2 ± 2.2 |
|  | group | 600 | 159 ± 8 | 309 ± 25 | 14.8 ± 2.3 | 25.0 ± 3.6 |
|  |  | 2000 | 158 ± 8 | 305 ± 26 | 15.1 ± 3.3 | 28.9 ± 3.3 |
|  | Satellite group | Control | 158 ± 17 | 348 ± 34 | 12.5 ± 2.7 | 23.0 ± 3.2 |
|  |  | 2000 | 159 ± 14 | 306 ± 36 | 12.7 ± 2.5 | 27.3 ± 3.6 |
|  |  |  |  |  |  |  |
| **Females** |  | Control | 186 ± 14 | 618 ± 75 | 21.4 ± 1.8 | 33.7 ± 2.2 |
|  | Treatment | 200 | 186 ± 16 | 610 ± 90 | 21.0 ± 1.8 | 30.1 ± 2.9 |
|  | group | 600 | 187 ± 12 | 599 ± 76 | 20.8 ± 1.9 | 32.3 ± 8.7 |
|  |  | 2000 | 186 ± 9 | 568 ± 63 | 20.3 ± 2.3 | 32.5 ± 3.7 |
|  | Satellite group | Control | 186 ± 9 | 605 ± 81 | 20.7 ± 1.8 | 34.2 ± 3.7 |
|  |  | 2000 | 186 ± 15 | 605 ± 35 | 20.7 ± 1.9 | 33.0 ± 3.3 |

The satellite group was given the vehicle control or Thai rice instant-granules containing turmeric extract and *Phyllanthus emblica* fruit pulp at 2,000 mg/kg BW daily for 180 days followed by no treatment for 28 days.

TR instant-granules: Thai rice instant-granules containing turmeric extract and *Phyllanthus emblica* fruit pulp

Values are expressed as mean ± SD.

**Table S3.** Histopathological findings observed in rats treated with 2,000 mg/kg BW of Thai rice instant-granules containing turmeric extract and *Phyllanthus emblica* fruit pulp granules in the chronic toxicity test.

| **Organs** | **Pathological changes** | **Main group** | | | |  | **Satellite group** | | | |
| --- | --- | --- | --- | --- | --- | --- | --- | --- | --- | --- |
|  |  | **Control** | | **2000 mg/kg** | |  | **Control** | | **2000 mg/kg** | |
|  |  | **M** | **F** | **M** | **F** |  | **M** | **F** | **M** | **F** |
| Brain | Intraneuronal inclusion | 0 | 0 | 0 | 0 |  | 0 | 0 | 0 | 0 |
|  | Chromatolysis | 0 | 0 | 0 | 0 |  | 0 | 0 | 0 | 0 |
|  | Acidophilic neuronal necrosis | 0 | 0 | 0 | 0 |  | 0 | 0 | 0 | 0 |
|  | Hippocampal neuronal loss | 0 | 0 | 0 | 0 |  | 0 | 0 | 0 | 0 |
|  | Neuronophagia | 0 | 0 | 0 | 0 |  | 0 | 0 | 0 | 0 |
|  | Gliosis | 0 | 0 | 0 | 0 |  | 0 | 0 | 0 | 0 |
|  | Perivascular cuffing | 0 | 0 | 0 | 0 |  | 0 | 0 | 0 | 0 |
|  | Hemorrhage | 0 | 0 | 0 | 0 |  | 0 | 0 | 0 | 0 |
| Heart | Myocardial calcification | 0 | 0 | 0 | 0 |  | 0 | 0 | 0 | 0 |
|  | Hydropic degeneration | 0 | 0 | 0 | 0 |  | 0 | 0 | 0 | 0 |
|  | Fatty degeneration | 0 | 0 | 0 | 0 |  | 0 | 0 | 0 | 0 |
|  | Myocarditis | 0 | 0 | **2** | 0 |  | 0 | 0 | **1** | 0 |
| Lung | Lymphoid proliferated peribronchioles | **1** | 0 | 0 | 0 |  | 0 | 0 | 0 | 0 |
|  | Pulmonary edema | 0 | 0 | 0 | 0 |  | 0 | 0 | 0 | 0 |
|  | Pulmonary hemorrhage | 0 | 0 | 0 | 0 |  | 0 | 0 | 0 | 0 |
|  | Foamy macrophage infiltration | 0 | 0 | 0 | 0 |  | **1** | **1** | 0 | 0 |
| Liver | Fatty degeneration | **3** | **1** | **7** | **3** |  | **1** | **2** | **2** | **1** |
|  | Hepatocyte megalocytosis | 0 | 0 | 0 | 0 |  | 0 | 0 | 0 | 0 |
|  | Lymphoid aggregated periportal area | **1** | **1** | 0 | 0 |  | 0 | 0 | 0 | 0 |
|  | Bile duct proliferation | **1** | 0 | 0 | **1** |  | 0 | **1** | 0 | **2** |
|  | Hepatitis/cholangiohepatitis | 0 | 0 | 0 | 0 |  | 0 | **1** | 0 | **1** |
|  | Oval cell hyperplasia | 0 | 0 | 0 | 0 |  | 0 | 0 | 0 | 0 |
| Kidney | Glomerulonephritis | 0 | 0 | 0 | 0 |  | 0 | 0 | 0 | 0 |
|  | Tubulonephrosis | 0 | 0 | 0 | 0 |  | 0 | 0 | 0 | 0 |
|  | Tubular cast | 0 | 0 | 0 | **1** |  | 0 | 0 | 0 | 0 |
|  | Renal infarction | 0 | 0 | 0 | 0 |  | 0 | 0 | 0 | 0 |
|  | Interstitial nephritis | **2** | **1** | **1** | **1** |  | 0 | 0 | **1** | 0 |
| Spleen | Lymphoid atrophy | 0 | 0 | 0 | 0 |  | 0 | 0 | 0 | 0 |
|  | Lymphoid hyperplasia | 0 | 0 | 0 | 0 |  | 0 | 0 | 0 | 0 |
|  | Inflammation | 0 | 0 | 0 | 0 |  | 0 | 0 | 0 | 0 |
| Thymus | Lymphoid atrophy | 0 | 0 | 0 | 0 |  | 0 | 0 | 0 | 0 |
|  | Lymphoid hyperplasia | 0 | 0 | 0 | 0 |  | 0 | 0 | 0 | 0 |
| Pancreas | Pancreatitis, fat necrosis | 0 | 0 | 0 | 0 |  | 0 | 0 | **1** | 0 |
|  | Adenocarcinoma of the pancreas | 0 | 0 | 0 | 0 |  | 0 | 0 | 0 | 0 |
|  | Pancreatic duct hyperplasia/tumor | 0 | 0 | 0 | 0 |  | 0 | 0 | 0 | **1** |
| Stomach | Squamous cell hyperplasia (fore stomach) | 0 | 0 | 0 | 0 |  | 0 | 0 | 0 | 0 |
|  | Squamous cell papilloma (fore stomach) | 0 | 0 | 0 | 0 |  | 0 | 0 | 0 | 0 |
|  | SCC (fore stomach) | 0 | 0 | 0 | 0 |  | 0 | 0 | 0 | 0 |
|  | Hyperplasia (glandular stomach) | 0 | 0 | 0 | 0 |  | 0 | 0 | 0 | 0 |
|  | Adenoma/adenocarcinoma (glandular stomach) | 0 | 0 | 0 | 0 |  | 0 | 0 | 0 | 0 |
|  | Mineralization | 0 | 0 | 0 | 0 |  | 0 | 0 | 0 | 0 |
|  | Dilated gland/glandular cyst | 0 | 0 | 0 | 0 |  | **1** | 0 | 0 | 0 |
| Adrenal gland | Cortical fatty degeneration | 0 | 0 | 0 | 0 |  | 0 | 0 | 0 | 0 |
|  | Adrenalitis | 0 | 0 | 0 | 0 |  | 0 | 0 | 0 | 0 |
|  | Adrenocortical atrophy | 0 | 0 | 0 | 0 |  | 0 | 0 | 0 | 0 |
|  | Nodular hyperplasia | 0 | 0 | 0 | 0 |  | 0 | 0 | 0 | 0 |
|  | Nodular adrenal medullary hyperplasia | 0 | 0 | 0 | 0 |  | 0 | 0 | 0 | 0 |
| Testis | Testicular hypoplasia | 0 | - | 0 | - |  | 0 | - | 0 | - |
|  | Testicular atrophy | 0 | - | 0 | - |  | 0 | - | 0 | - |
|  | Orchitis | 0 | - | 0 | - |  | 0 | - | 0 | - |
|  | Testicular tumor | 0 | - | 0 | - |  | 0 | - | 0 | - |
| Epididymis | Epididymitis | 0 | - | 0 | - |  | 0 | - | 0 | - |
| Seminal vesicle | Seminal vesiculitis | 0 | - | 0 | - |  | 0 | - | 0 | - |
| Prostate | Prostatic hyperplasia | 0 | - | 0 | - |  | 0 | - | 0 | - |
|  | Prostate tumor | 0 | - | 0 | - |  | 0 | - | 0 | - |
|  | Prostatitis | 0 | - | 0 | - |  | 0 | - | 0 | - |
| Ovary | Oophoritis | - | 0 | - | 0 |  | - | 0 | - | 0 |
|  | Ovarian degeneration | - | 0 | - | 0 |  | - | 0 | - | 0 |
| Uterus | Inflammation | - | 0 | - | 0 |  | - | 0 | - | 0 |
|  | Hyperplasia | - | **2** | - | 0 |  | - | 0 | - | 0 |
|  | Polyps | - | 0 | - | 0 |  | - | 0 | - | 0 |
|  | Neoplasm | - | 0 | - | 0 |  | - | 0 | - | 0 |

A satellite group was given the vehicle control or Thai rice instant granules containing turmeric extract and *Phyllanthus emblica* fruit pulp at 2,000 mg/kg BW daily for 180 days followed by no treatment for 28 days.

The specific number represents the number of abnormal animals. n = 10 per sex for the main group and n = 5 for the satellite groups. M: Male, F: Female


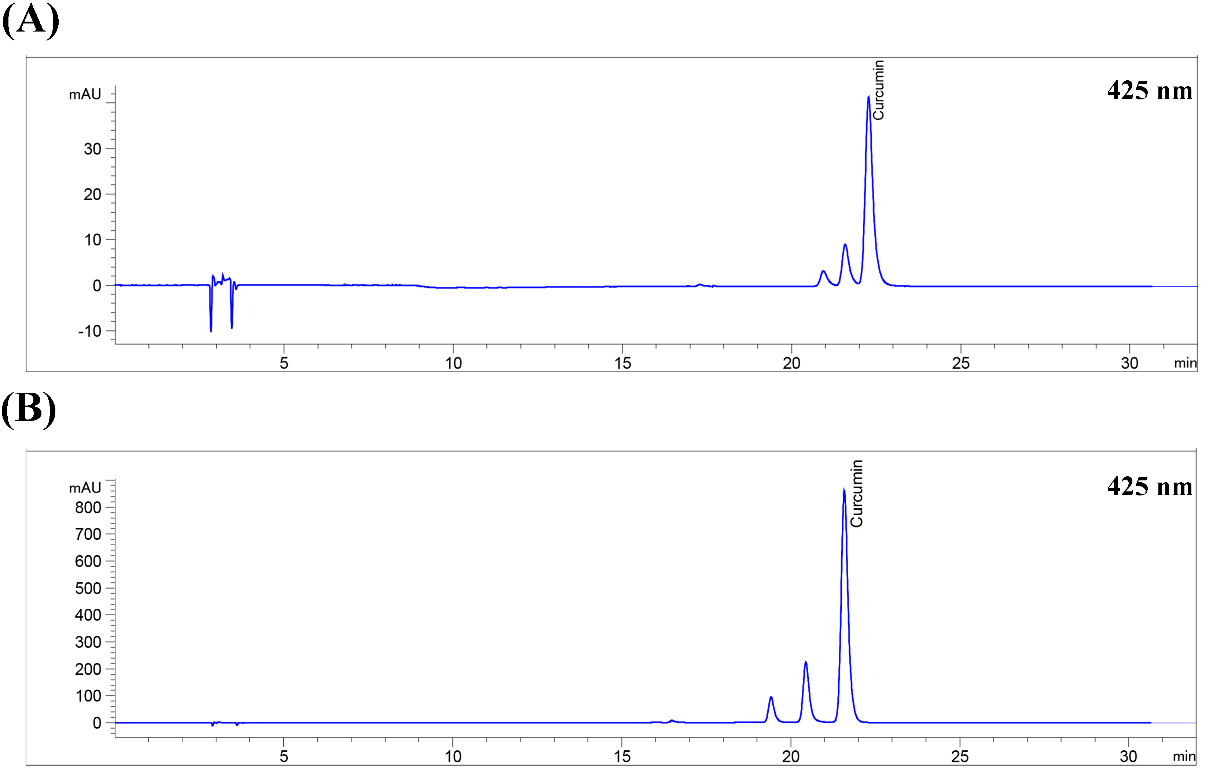


**Figure S1.** Representative chromatograms of (A) curcumin standard (10 μg/ml) and (B) Thai rice instant granules (1000 μg/ml), obtained at a detection wavelength of 425 nm.
